# Ideal Length of Oral Endotracheal Tube for Critically Ill Intubated Patients in an Asian Population: Comparison to Current Western Standards

**DOI:** 10.7759/cureus.3590

**Published:** 2018-11-14

**Authors:** Amos Lal, Eleanor D Pena, Dizon J Sarcilla, Peter P Perez, Johnny C Wong, Faheem A Khan

**Affiliations:** 1 Internal Medicine, Saint Vincent Hospital, Worcester, USA; 2 Internal Medicine, Ng Teng Fong General Hospital, Jurong East, SGP

**Keywords:** intubated patient, oral endotracheal tube, trachea, western population, critical care, endotracheal intubation, asian population

## Abstract

Background

Endotracheal (ET) intubation is used to maintain the airway patency of patients during mechanical ventilation and is inserted at a particular depth into the trachea through the nose, mouth, or through an incision in the neck. The aim of our study was to validate the ideal length of an oral endotracheal tube (ETT) in the Asian population compared to Western standards.

Methods

Patient records with an oral ETT inserted between April 2011 and June 2015 in the Intensive Care Unit (ICU) of a hospital were retrospectively analyzed. The key variables included demographics, height, and ideal body weight of the patient, length of the oral ETT, and chest X-rays. Statistical analyses were performed using R software (https://cran.r-project.org/).

Results

There were 876 incidences of oral cuffed ETT insertions in 708 adult patients ≥ 18 years of age. The median ETT depth in all the ethnic groups (Chinese, Malay, Indians, and others) was 22 cm. The median depth of oral ETTs was 22 cm in males and 21 cm in females as compared to Western standards (males: P < 0.0001; females: P = 0.93). In ICU patients intubated with an ETT at an acceptable distance from the carina (2 - 5 cm), the median ETT depth was different in males (P < 0.0001) but was similar in females (P = 0.87).

Conclusion

We suggest that males and females in the Asian population, especially in South East Asia, should have their ETTs secured at the corner of mouth by at least 1 cm less in comparison to the Western population (22 cm in males and 20 cm in females).

## Introduction

The inappropriate depth of an endotracheal tube (ETT) can lead to trauma, sympathetic stimulation, recurrent laryngeal nerve compression, and unilateral intubation causing complications [1-2]. According to the American Society of Anesthesiologists Closed Claims Project, 2% of the adverse respiratory claims in adults and 4% in children were due to endobronchial intubation [3-4].

It becomes challenging in the remaining cases (27%) when the vocal cords are partially visible or invisible [5]. The most often recommended depth of placement of an ETT is 23 cm in males and 21 cm in females [6-7]. The conventional and current practice of securing an ETT at 23 cm in men and at 21 cm in women was developed in the United States (US); it has not been well studied in patients with non-Caucasian ethnicity. Therefore, in the present study, we analyzed the depth of oral ETT fixation in an Asian population in terms of gender and ethnicity and compared the observed differences against the Western standards.

## Materials and methods

Study design and setting

We analyzed the records of patients who had an oral ETT inserted in the Intensive Care Unit (ICU) of a restructured public hospital between April 2011 and June 2015.

Participants

All patients who were aged 18 and above, underwent oral ET intubation (via direct laryngoscopy or video laryngoscopy), and admitted in the ICU for any indication during the study period were eligible.

Variables and measurement

The records contained information, such as age, gender, height, and ideal body weight of the patient, the length of the oral ETT, and chest X-rays. The study included Asian patients from different ethnicities, such as Chinese, Indian, Malay, and others.

Both male and female populations were independently analyzed to calculate the days of intubation, length of stay (LOS), and ETT depth. The populations of both the genders were cumulatively analyzed for variations in ETT depth based on ethnicity (Chinese, Indian, Malay, and others) and indications for intubation (respiratory failure, collapse, postoperative, others). Studying the indications for intubation is pertinent since physicians from various specialties performing intubations face certain conditions more commonly as compared to other settings. Primary data sources for establishing the study cohort were patient records from the ICU. Chest x-ray (CXR) information was used to confirm the position of the ETT. Acceptable ETT positioning was defined as being placed at a distance 2 - 5 cm from the carina, whereas distances > 5 cm, < 2 cm, or in the right/left main bronchus were classified as “dangerous”.

Most of the patients intubated for respiratory failure were in the ICU and emergency room (ER) and thus intubated by ICU and ER physicians. Patients with collapse were intubated in the ER by ER physicians, whereas postoperative patients looked after in the ICU were intubated in the operating room by anesthesiologists. Of these, incidences with ETT depth in the acceptable range (2 - 5 cm) were included for the subgroup analysis of median ETT depth for the various indications of intubations.

Statistical methods

Statistical analyses were performed using R software (https://cran.r-project.org/). Continuous measurements were reported as means and standard deviations (SD) or medians and interquartile range (IQR) for measurements with skewed distributions. Categorical measurements were reported as counts and percentages. The two-sample t-test would be used to compare each of the gender groups against the Western standards or Wilcoxon signed-rank test if the samples violated the normality assumption. Statistical significance was determined at P < 0.05.

## Results

Initially, 950 patient records were included between April 2011 and June 2015. Among the 950 records, 74 records had missing information on height, weight, length of the oral ETT, and ETT position from the carina and were thus removed from the analysis. As such, the number of analyzable records was 876, in which there were 708 unique patients at or above the age of 18 years old. From our records, the top reason for intubation in patients was a respiratory failure (34.6%), followed by postoperative cases (32.1%), and collapse (11.8%). The remaining records (21.7%) were classified as others (i.e., none of the three reasons mentioned earlier). The demographic characteristics of the patients are shown in Table 1.

**Table 1:** Patient Demographics n: number: SD: standard deviation

Demographics	Male (n = 450)	Female (n = 258)
Mean age (SD) in years	62.7 (16.4)	67.5 (15.5)
Mean height (SD) in cm	166.8 (8.7)	154.4 (7.5)
Race (%)		
Chinese	308 (68.4%)	164 (63.6%)
Malay	57 (12.7%)	41 (15.9%)
Indian	40 (8.9%)	29 (11.2%)
Others	45 (10.0%)	24 (9.3%)

Out of the 708 unique patients, 450 (63.6%) were males and 258 (36.4%) were females. After stratifying by gender, the ethnic composition was similar in both groups; it was comprised mainly of the Chinese population (males, 68.4%; females 63.6%), followed by Malay (males, 12.7%; females 15.9%), Indian (males, 8.9%; females 11.2%), and others (males, 10.0%; females 9.3%). The mean age was 62.7 years (SD = 16.4) and 67.5 years (SD = 15.5) for males and females, respectively. The mean height was 166.8 cm (SD = 8.7) and 154.4 cm (SD = 7.5) for males and females, respectively.

**Figure 1:**
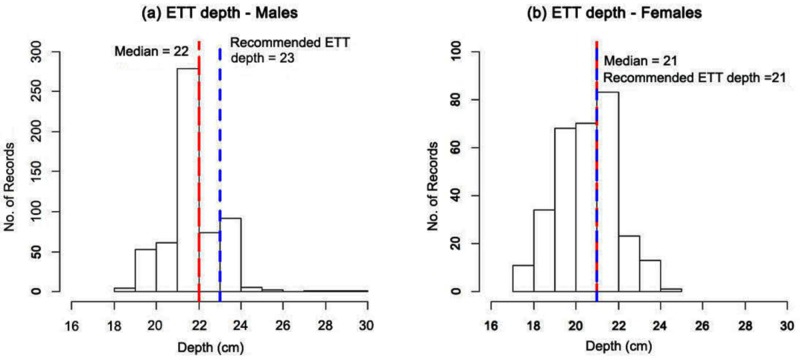
Median endotracheal tube (ETT) depth in both genders Median endotracheal tube depth in both genders: (a) males and (b) females. X-axis: ETT depth (cm); Y-axis: number of patient records. The red line represents the median depth. The blue dash line represents the recommended ETT depth (cm).

The median duration of intubation was two days (IQR: 2 - 4) for both gender groups, whereas the median length of stay (LOS) was four days (IQR: 2 - 7) for males and three days (IQR: 2 - 6) for females. The median ETT depth was 22 cm for males (IQR: 22 - 23) (Figure 1A), 21 cm for females (IQR: 20 - 22) (Figure 1B), and 22 cm for all the ethnic groups (IQR: 21 - 22 (Chinese), 21 - 22 (Malay), 20 - 22 (Indian), and 21 - 23 (others)). Furthermore, the median ETT depth for various indications of intubations (respiratory failure, postoperative cases, collapse, and others) was 22 cm.

In all patient records, more than half of the incidences with ETT depth were placed in the acceptable range of 2 - 5 cm from the carina (58.7%), followed by > 5 cm (26.6%), < 2 cm (12.4%), and in the right/left main bronchus (2.3%). The breakdown of the ETT tube position (distance from carina) for each of the indications for intubation is shown in Table 2.

**Table 2:** Breakdown of Patients for Each Indication of Intubation L: left; n: number; R: right

	Indications of intubation			
Distance from carina	Respiratory failure (n = 303)	Postoperative (n = 281)	Collapse (n = 103)	Others (n = 189)
< 2 cm	36 (11.9%)	32 (11.4%)	15 (14.6%)	26 (13.8%)
2 cm - 5 cm (Acceptable range)	184 (60.7%)	161 (57.3%)	62 (60.2%)	107 (56.6%)
> 5 cm	75 (24.8%)	83 (29.5%)	23 (22.3%)	52 (27.5%)
R / L main bronchus	8 (2.6%)	5 (1.8%)	3 (2.9%)	4 (2.1%)

The difference in the total number of patients (male and female) in Table 1 and Table 2 is different because Table 1 shows details about unique patients, while Table 2 includes numbers for the patient encounter (some patients were re-admitted to the ICU during the same hospital stay).

Percentages are expressed in terms of the breakdown of the distance from the carina for each indication of intubation (right/left).

Additionally, the subgroup analysis for the acceptable range showed that the median ETT depth was 22 cm (IQR: 21 - 22).

**Figure 2:**
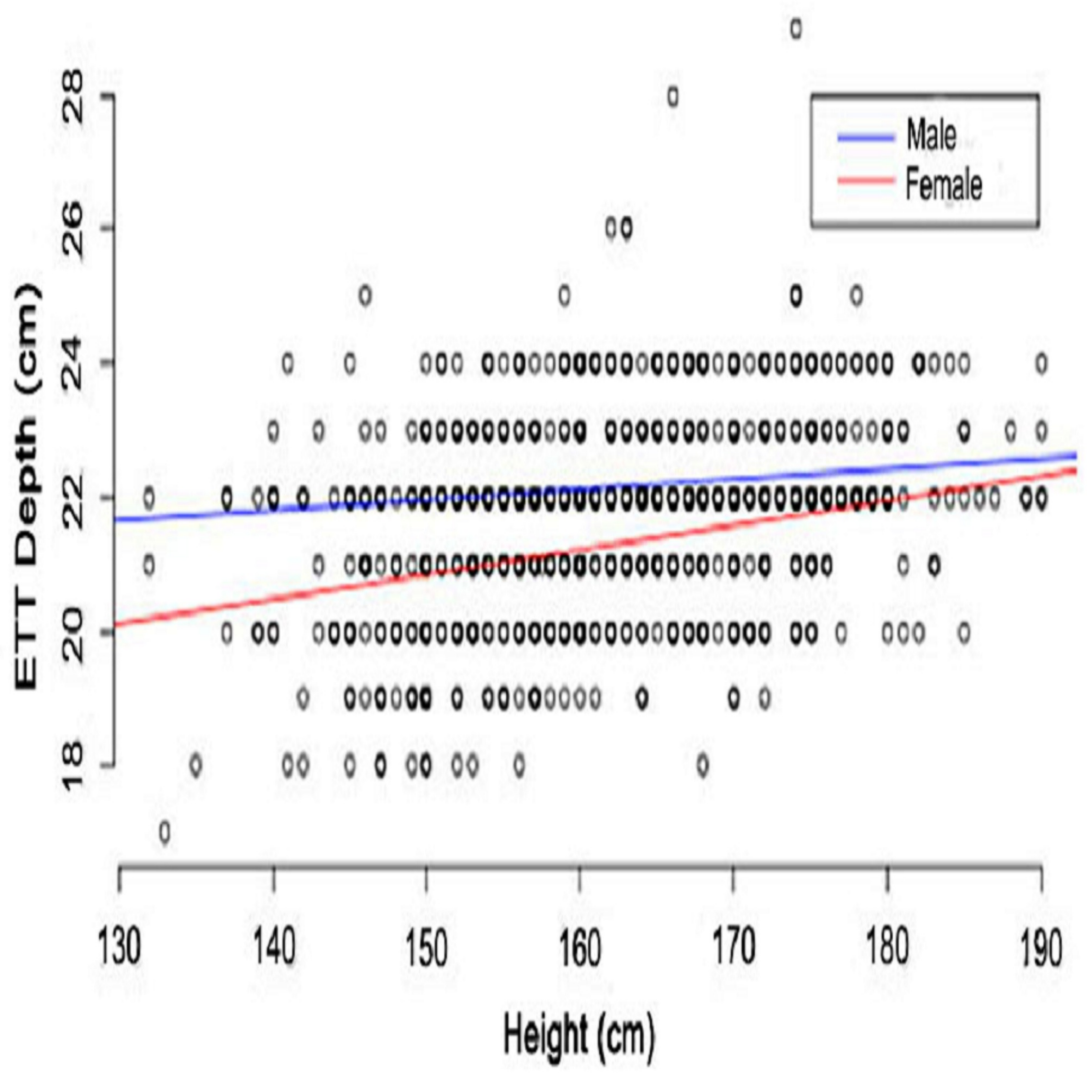
Relationship between endotracheal tube (ETT) depth and height by gender X-axis: ETT depth (cm); Y-axis: height of study population (cm). The red and blue lines represent the male and female populations, respectively. The bold points represent multiple patients having the same set of data points.

**Figure 3:**
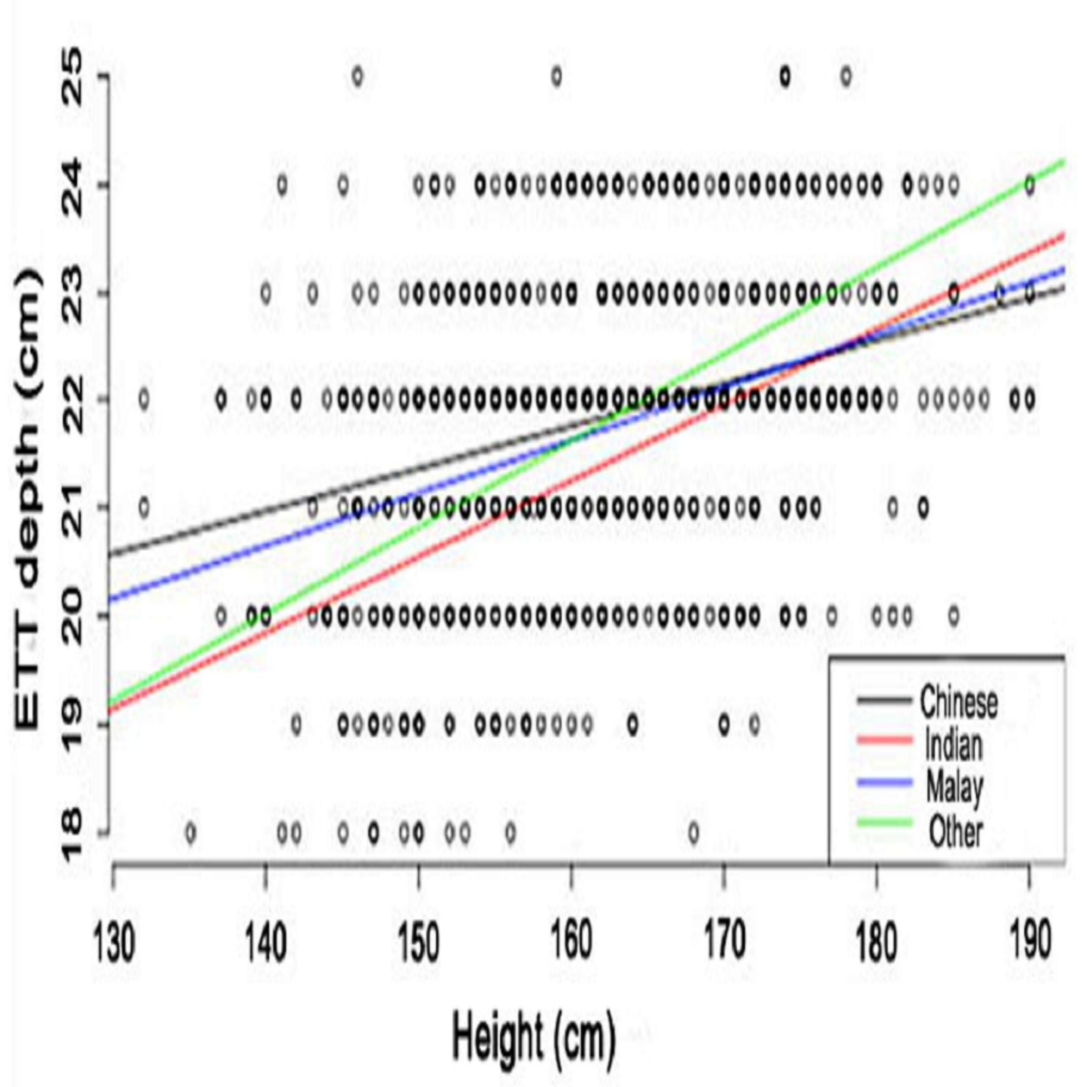
Relationship between endotracheal tube (ETT) depth and height by ethnicity X-axis: height of study population (cm); Y-axis: ETT depth (cm). The black, red, blue, and green lines represent the Chinese, Indian, Malay, and Other populations, respectively. The bold points represent multiple patients having the same set of data points.

Figure 2 shows the linear relationship between the ETT depth and patient height. The correlation was observed for both male and female patients; however, the slope varied when compared between the genders. Furthermore, the slope varied for different ethnicities, and a linear correlation was seen (Figure 3). The depth of the ETT increased with the height of the individual. At the height of 130 - 160 cm, the Chinese population showed a maximum ETT depth followed by Malay, Others, and Indian populations. The differences between the populations decreased at a height of 160 - 170 cm and followed a substantial difference at a height of 190 cm, in which other ethnic groups exhibited a maximum ETT depth followed by Indian, Malay, and Chinese populations.

 Table 3 shows a comparison of the median ETT depth between Intensive Care Unit patients and the ETT depth guideline.

**Table 3:** Comparison of Median ETT Depth Between Intensive Care Unit Patients and ETT Depth Guideline The counts for each gender group refers to the number of records; P-value^: used the Wilcoxon signed-rank test ETT: endotracheal tube; n: number

	Median ETT depth (cm)	ETT depth in Western standards (cm)	P-value^
Main analysis*			
Males (n = 573)	22	23	< 0.0001
Females (n = 303)	21	21	0.93
Subgroup analysis* (look at patients with acceptable ETT position)			
Males (n = 320)	22	23	< 0.0001
Females (n = 194)	21	21	0.87

The Wilcoxon signed-rank test for median ETT depth in the Asian male population showed strong evidence against the Western standards of 23 cm (P < 0.0001). On the other hand, there was no evidence of the median ETT depth in the Asian female population being different from the Western standards of 21 cm (P = 0.93). Similarly, a subgroup analysis of the median ETT depth in patients with an ETT at an acceptable distance from the carina (2 - 5 cm) revealed strong evidence against the Western standards among Asian males (P < 0.0001) but no evidence against the Western standards among the Asian females (P = 0.87).

## Discussion

Safe placement of an ETT in patients is of prime importance. It has been well documented that the ETT moves downwards toward the carina by at least 2 cm in flexion of the neck and upwards by 2 cm in extension. This plays an important role when securing ETTs, especially in intensive care patients, as they tend to be intubated occasionally for a prolonged period of time and require a lot of movements during day-to-day care. Securing the tube too low or too high can cause either right-sided endobronchial intubation or a high risk of accidental extubation, respectively. These inadvertent impediments predispose already critically ill patients to significant risks and complications. In 2005, failure to identify the incorrect placement of an ETT was the foremost cause of hypoxemia and death during general anesthesia [8]. A radiographic confirmation of the correct placement is of the utmost importance since it helps to rule out other causes of respiratory failure as well [9].

The standard depth of placing an ETT has been determined in the Caucasian population to be 23 cm and 21 cm for males and females, respectively [6]. However, determining the depth of the ETT using conventional methods is controversial. Sitzwohl et al. suggested that the depth of the ET intubation by conventional methods was very deep and recommended that the depth from the central incisor should be 22 cm in males and 20 cm in females [10]. Another study by Ong et al. reported that the use of 23/21 rule in Asian populations increases the risk of main stem intubation [11]. This could be because Asians are generally of shorter stature in comparison to the Western population, especially Caucasians. Various studies have reported on the safe ETT depth in different Asian populations. Park et al. reported that ETT fixation is safe at 20 cm in Korean males and at 18 cm in females [12], whereas Varshney et al. reported that the ETT depth in the Indian population was 20.26 cm and 18.23 cm in males and females, respectively, from the right corner of the mouth [2].

In the present study, we analyzed the median ETT depth for ICU patients in the Asian population. The local population in Singapore is representative of three major ethnic groups (Malay, Chinese, and Indian), which covers almost two-thirds of the population of Asia. The aim of the current investigation was to determine the depth of the ETT in the Asian population and compare it with Western standards. We analyzed records of patients ≥ 18 years of age and found that the median ETT depth in Asian males and females was 22 cm and 21 cm, respectively (Figure 1). However, the median depth of the ETT intubation, irrespective of the gender, was the same in all the ethnic populations (Chinese, Indian, Malay, Others). In our study, we also found that the depth of intubation was directly related to the height of the individual, and there was a linear correlation in both male and female populations across all ethnicities (Figure 3). Furthermore, the slope of correlation varied with various ethnic groups; for example, other racial subgroups had a steeper slope curve as compared to the Chinese or Asian populations which exhibited a flatter slope.

Chest x-rays demonstrated that 514 (58.7%) of the 876 intubations were placed in the acceptable range (2 - 5 cm), while inappropriate placements (< 2 cm, > 5 cm) were detected in 109 (12.4%) and 233 (26.6%) intubations, respectively. Endobronchial intubations (right/left bronchus) occurred in 20 (2.3%) patients. These results are consistent with a study by Han et al. which highlighted that routine oral ET intubation in the operating theater cannot always assure optimal ETT depth [13].

A few studies have also reported a significant correlation between the ETT length and the height of the patient [14-16]. Nafisi et al. reported that even though men, in general, are taller than women and the ETT needs to be fixed deeper in them, the ETT might be placed deeper in men than women even when they have the same body height [17]. In our study, we observed that the relationship between the ETT depth and the patient's height is affected by gender, but it may not be of clinical importance except in very extreme cases (Figure 3). For instance, the maximum difference in the ETT depth was seen at the height of 130 cm, whereas the difference was minimized at 190 cm. Thus, it could be inferred that people with a shorter height require a more cautious insertion regarding the ETT depth.

Additionally, the median depth of the ETT in the male population was found to be significantly different from the Western standards, but not in females. The median ETT depth at an acceptable distance from the carina in the study groups was also significantly different than Western standards in males, but not in females (Table 3).

The study had a few limitations. Firstly, the patient records of only adults of age ≥ 18 years were analyzed. Hence, the results of this study are not applicable to the population of lesser age, even if they are lying in the same height range. Also, the study results are not applicable to patients with unusual height, i.e., beyond the range studied. Few studies have also reported that patients with short neck length face difficulty in safe ETT intubation; hence, our results are not applicable to this population [18]. A further limitation was the difference in the number of males and females included in the study (63.6% vs. 36.4%). A few studies have reported that females are at higher risk of endobronchial intubation, and this might have contributed to lesser patient records with misplaced ETTs and affected the results [10-11].

This work was presented at the ESICM LIVES 2016 Meeting, Milan, Italy, October 1-5, 2016 (Abstract A655 - Lal A, Khan FA, Dela Pena EG, Dizon JS, Perez PPP, Wong CMJ: Ideal length of the oral endotracheal tube (ETT) for intubated patients in the Asian population: comparison to current Western standards). 

## Conclusions

Based on the observations made in our study, we suggest that males and females in the Asian population, especially in South East Asia, should have their endotracheal tubes secured at the corner of mouth by at least 1 cm less in comparison to the Western population. Although waveform capnography can differentiate between endotracheal intubation from esophageal intubation, a chest x-ray should be performed to confirm the position of the ETT, regardless of the indication, place of intubation, or the personnel involved in the procedure.
